# Increased risk of impaired treatment satisfaction among girls/women and young people with suboptimal HbA1c: Results of a nationwide type 1 diabetes study

**DOI:** 10.1186/s13098-021-00673-0

**Published:** 2021-05-19

**Authors:** Anna Stahl-Pehe, Silvia Selinski, Christina Bächle, Joachim Rosenbauer

**Affiliations:** 1 Institute for Biometrics and Epidemiology, German Diabetes Center, Leibniz Center for Diabetes Research, Auf’m Hennekamp 65, 40225 Düsseldorf, Germany; 2 German Center for Diabetes Research (DZD), Munich-Neuherberg, Germany

**Keywords:** Type 1 diabetes mellitus, Epidemiology, Patient-reported outcome

## Abstract

**Background:**

This study aims to analyze the patient-reported outcome (PRO) of treatment satisfaction in a sample of children, adolescents and young adults with long-duration type 1 diabetes and to determine potential risk factors for poor treatment satisfaction and the intraindividual changes over a 3-year period.

**Methods:**

This study used data from two population-based questionnaire surveys conducted in 2015–2016 and 2018–2019. The participants were 11 to 27 years old and had a type 1 diabetes duration of 10 years or longer in 2015–2016 (n = 575). Factors that were potentially associated with poor treatment satisfaction (moderate, poor or very poor) compared to the reference group (very good or good treatment satisfaction) were analyzed by log binomial regression adjusted for sex and age group.

**Results:**

In 2015–2016 (2018–2019), 26% (33%) of the respondents rated their diabetes treatment/consultation as "very good", 53% (46%) as "good", and 20% (21%) as "poor". Based on the 2018–2019 data, girls/women had an increased risk of poor treatment satisfaction (RR_girls/women_: 1.64 (1.10; 2.44), p = 0.016). In addition, people with hemoglobin A1c (HbA1c) values ≥ 7.5% had a more than twice the risk of poor treatment satisfaction than people with HbA1c values < 7.5% (RR_HbA1c ≥7.5%_: 2.43 (1.63; 3.63), p < 0.001). A total of 42% of people with poor treatment satisfaction in 2015–2016 also reported poor treatment satisfaction at follow-up.

**Conclusions:**

Most study participants were satisfied with their diabetes treatment. However, we identified risk groups that would benefit from targeted interventions to improve this important PRO.

**Supplementary Information:**

The online version contains supplementary material available at 10.1186/s13098-021-00673-0.

## Background

Treatment satisfaction is a patient-reported outcome that may be helpful for understanding the patient's perspective on his or her current treatment [1]. Treatment satisfaction is defined as the patient's subjective assessment of the treatment experience. It includes both the process and the results of the treatment experience [2, 3] and comprises an assessment of the individual’s needs, perceived benefits, concerns, and expectations [4, 5]. It is clinically relevant to assess the extent to which patients are satisfied with health care because treatment satisfaction plays an important role in treatment success [1, 6, 7]. Treatment satisfaction is an important factor in quality of care, especially in the treatment of chronic diseases such as diabetes mellitus. In patients with diabetes, lower treatment satisfaction leads to greater difficulties in adhering to treatment [8]. Furthermore, one of the factors most likely to affect self-care adherence may be treatment satisfaction [7]. This means that the identification of parameters that independently influence treatment satisfaction may contribute to improved clinical outcomes [8].

Factors influencing treatment satisfaction continue to be the subject of research and are largely unexplored in children, adolescents and young adults with type 1 diabetes. The aim of the following analysis is to describe subjective satisfaction with treatment in a group of children, adolescents and young adults with a long duration (≥ 10 years) of type 1 diabetes and to examine potentially influencing factors and intraindividual changes over time. Our hypothesis is that both sociodemographic factors and diabetes-related factors are related to treatment satisfaction.

## Methods

**Fig. 1:**
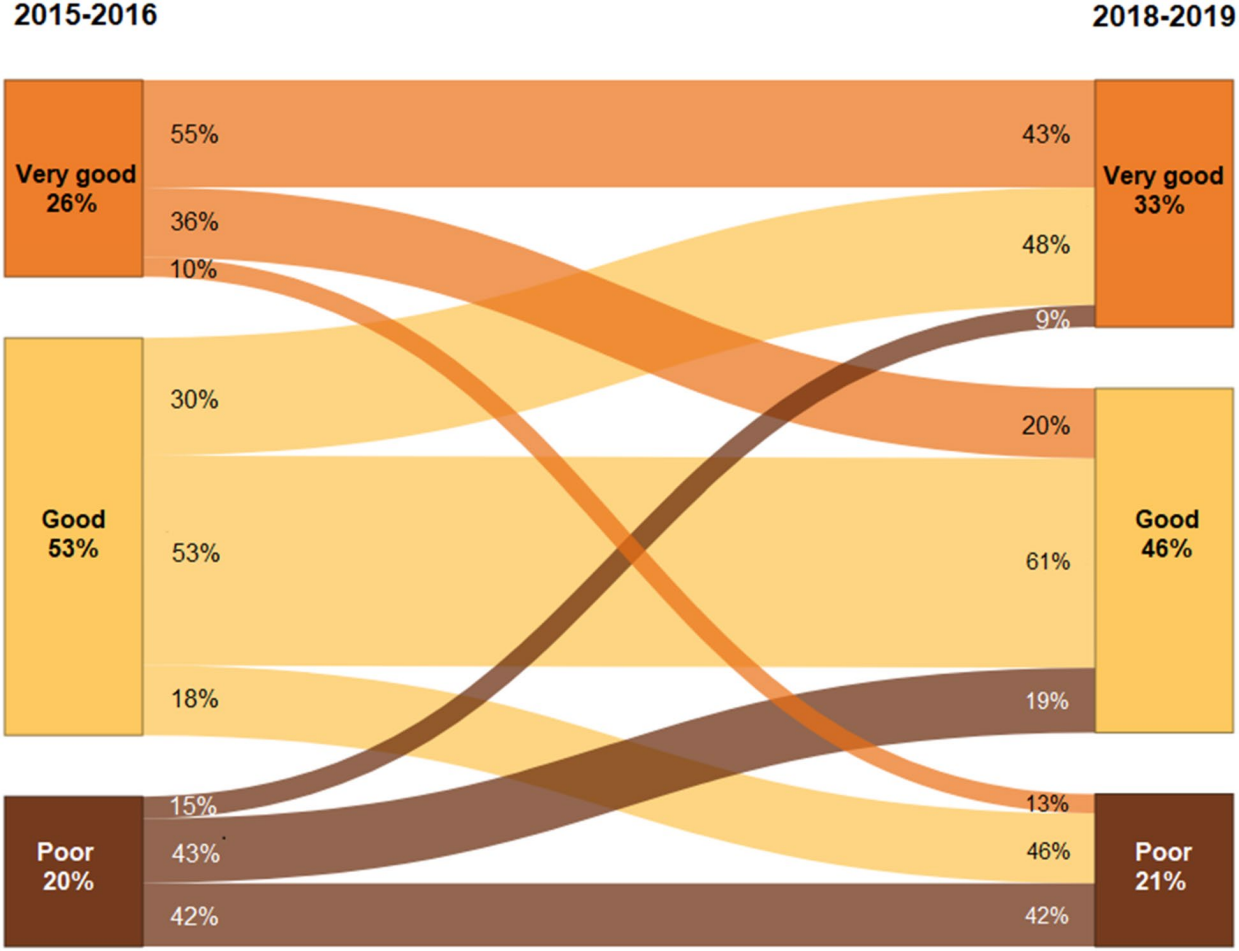
Sankey diagram of intraindividual changes in treatment satisfaction over a 3-year period. The possible answers “moderate”, “poor” and “very poor” were summarized as “poor”. The width of the arrows shown is proportional to the relative frequency

As part of a Germany-wide epidemiological longitudinal study that has been carried out since 2009, children from the age of 11 years, adolescents and adults with early-onset type 1 diabetes (i.e., with a diagnosis before the age of 5 years) and a diabetes duration of at least 10 years were comprehensively surveyed [9, 10]. Physicians diagnosed type 1 diabetes according to the International Society for Pediatric and Adolescent Diabetes (ISPAD) guidelines [11]. The participants answered the question “How do you currently rate your diabetes treatment/consultation overall?” to assess treatment satisfaction. Responses were given on a 5-point Likert scale (very good, good, moderate, poor, very poor). We evaluated self-reported data from the two most recently conducted surveys. In total, 1133 and 719 people participated in the 2015–2016 and 2018–2019 surveys, respectively; of these, 97% (1097 and 697 people) answered the question on treatment satisfaction. A total of 587 people took part in both surveys. We analyzed the data from 575 people who answered the question about treatment satisfaction in both surveys. We characterized the sample by group percentage or the mean and standard deviation (SD) (variables in Table 1). Groups were compared using the likelihood ratio (LR) and Wald tests in a log binomial model adjusted for sex and age group. The change in treatment satisfaction between the two surveys was visualized with a Sankey diagram (Fig. 1 and Additional file 1: Figures S1–S6).

**Table 1:** Sample characteristics for the total sample and stratified according to treatment satisfaction (survey 2018–2019)

Characteristic	Treatment satisfaction									p-value^b^
	Total		Very good			Good		Poor^a^		
	% or mean (SD)	N	% or mean (SD)	N		% or mean (SD)	N	% or mean (SD)	N	
Sex										0.002
Male	41%	238	46%	88		44%	117	28%	33	
Female	59%	337	54%	103		56%	149	72%	85	
Age [years]	20.9 (4.0)	575	20.5 (3.9)	191		20.9 (3.8)	266	21.4 (4.4)	118	0.002
Age group										0.086
14–17 years	26%	151	27%	52		25%	66	28%	33	Ref.
18–21 years	38%	221	42%	81		40%	106	29%	34	0.062
22–25 years	21%	121	18%	34		22%	59	24%	28	0.888
26–30 years	14%	82	13%	24		13%	35	19%	23	0.499
Socioeconomic status										0.606
Low	16%	90	16%	30		16%	43	14%	17	Ref.
Intermediate	45%	258	44%	84		43%	114	51%	60	0.479
High	39%	225	39%	75		41%	109	35%	41	0.805
Unknown	0%	2	1%	2		0%	0	0%	0	1.000
Age at manifestation [years]	2.9 (1.2)	575	2.9 (1.2)	191		2.9 (1.1)	266	2.9 (1.2)	118	0.607
Diabetes duration [years]	18.0 (3.7)	575	17.6 (3.6)	191		18.0 (3.7)	266	18.5 (4.1)	118	0.493
BMI-SDS	0.37 (0.95)	568	0.40 (0.90)	189		0.31 (0.99)	264	0.44 (0.95)	115	0.613
HbA1c [mmol/mol]	61.6 (14.9)	560	57.3 (10.8)	188		62.2 (15.4)	258	67.3 (17.4)	114	0.027
HbA1c [%]	7.8 (1.3)	560	7.4 (1.0)	188		7.8 (1.4)	258	8.2 (1.3)	114	< 0.001
< 7.5% (< 58 mmol/mol)	46%	267	60%	115		46%	122	25%	30	Ref.
≥ 7.5% (≥ 58 mmol/mol)	51%	293	38%	73		52%	136	71%	84	< 0.001
Unknown	3%	15	2%	3		3%	8	3%	4	0.060
Insulin pump										0.101
No	29%	165	22%	42		33%	87	31%	36	Ref.
Yes	71%	409	78%	149		67%	178	69%	82	0.037
Unknown	0%	1	0%	0		0%	1	0%	0	1.000
Glucose sensor^c^										0.303
No, never	18%	106	18%	35		18%	48	19%	23	Ref.
Yes, temporarily	17%	99	17%	33		15%	40	22%	26	0.632
Yes, permanently	64%	369	64%	123		67%	177	58%	69	0.261
Unknown	0%	1	0%	0		0%	1	0%	0	1.000
Participation in the Type 1 Diabetes Disease Management Program										0.329
Yes	45%	260	48%	92		44%	117	43%	51	Ref.
No	32%	185	29%	55		32%	84	39%	46	0.089
Don´t know	22%	124	22%	42		23%	61	18%	21	0.986
Unknown	1%	6	1%	2		2%	4	0%	0	1.000
Hypoglycemia with hospitalization during the past 12 months										0.960
No	97%	560	98%	187		97%	258	97%	115	
Yes	3%	15	2%	4		3%	8	3%	3	
Diabetic ketoacidosis with hospitalization during the past 12 months										0.471
No	95%	548	97%	185		95%	252	94%	111	
Yes	5%	27	3%	6		5%	14	6%	7	
Diabetes-related long-term complications										0.527
No	78%	446	80%	153		76%	201	78%	92	Ref.
Yes	17%	98	17%	32		18%	48	15%	18	0.324
Unknown	5%	31	3%	6		6%	17	7%	8	0.676

^a^The possible answers “moderate”, “poor” and “very poor” were grouped

^b^p-value according to likelihood ratio test (global) or Wald test for categorical variables (comparison with reference) adjusted for sex and age group

^c^Including both continuous glucose monitoring (CGM) and flash glucose monitoring (FGM)

Factors that were potentially associated with poor/impaired treatment satisfaction (moderate, poor, and very poor) compared to the reference group (which reported very good or good treatment satisfaction) were analyzed by log binomial regression adjusted for sex and age group. Forward variable selection using the Akaike information criterion (AIC) and LR tests yielded the final regression model for the first and second surveys. The variables considered for the selection procedure are shown in Table 1. The final regression model for the 2018–2019 survey included sex, age group and hemoglobin A1c (HbA1c) group. To analyze the differential effects of sex by HbA1c level and vice versa we additionally included a sex by HbA1c level interaction term in the regression model. Furthermore, we included the treatment satisfaction reported in the previous survey in the final model. We report the relative risks (RRs), 95% confidence intervals (95% CIs), and LR and Wald test p-values for the risk of impaired treatment satisfaction.

## Results

The study participants are characterized in Table 1. The very good, good and poor treatment satisfaction groups differed in terms of sex ratio, age and HbA1c value. Similar levels of treatment satisfaction were reported in both periods. In 2015–2016 (2018–2019), 26.4% (33.2%) of the respondents rated their diabetes treatment/consultation as "very good", 53.4% (46.3%) as "good", 16.5% (17.2%) as "moderate", 3.0% (2.6%) as "poor" and 0.7% (0.7%) as "very poor". Based on the 2018–2019 data, girls/women had a 64% higher risk of impaired (moderate, poor or very poor) treatment satisfaction than boys/men (RR _girls/women_: 1.64 (1.10; 2.44), p = 0.016). People with HbA1c values ≥ 7.5% had a more than twice the risk of impaired treatment satisfaction compared to people with HbA1c values < 7.5% (RR _HbA1c ≥7.5%_: 2.43 (1.63; 3.63), p < 0.001). The risk of impaired treatment satisfaction did not differ according to age group (for details see Additional file 1: Table S1). The strength of the finding that girls/women were more likely to be dissatisfied with care than boys/men was independent of HbA1c level in 2018–2019. The finding that patients with suboptimal HbA1c levels were more likely to be dissatisfied with care than patients with controlled HbA1c levels was equally strong in female and male persons (p_sex_ = 0.002, p_HbA1c_ < 0.001, p_sex*HbA1c_ = 0.332, p_age group_ = 0.087).

Figure 1 shows the distribution of treatment satisfaction among all participants at both survey times. Forty-two percent of people who had poor treatment satisfaction in 2015–2016 also reported poor treatment satisfaction 3 years later. Of the people with very good treatment satisfaction in 2015–2016, 10% were dissatisfied with their treatment 3 years later. Considering differences in sex, age and HbA1c value, patients who had poor treatment satisfaction in 2015–2016 had a relative risk of 2.8 for equally impaired treatment satisfaction in 2018–2019 (RR _poor treatment satisfaction in 2015–2016_: 2.77 (1.96; 3.92), p < 0.001) (Additional file 1: Table S2).

## Discussion

For the first time, this study provides data on treatment satisfaction among young people with type 1 diabetes in Germany. Approximately 4 out of 5 of the surveyed children, adolescents and adults with long-duration diabetes rated their diabetes treatment and consultation as very good or good at both survey times. In the second survey, the risk of impaired treatment satisfaction was increased in girls/women and people with suboptimal blood sugar control. In addition, impaired treatment satisfaction in the first survey was a predictor of impaired treatment satisfaction 3 years later. In a recent Swedish study with 138 15- to 20-year-olds with type 1 diabetes, a lower level of treatment satisfaction was also observed in people with high HbA1c values (> 8.5% (> 69 mmol/mol)) than in people with more favorable HbA1c values. Treatment satisfaction did not differ between girls and boys in that study [12]. In a study with 108 14- to 18-year-olds with type 1 diabetes in England, treatment satisfaction was stable over the 3-year study period. In that sample, treatment satisfaction and HbA1c were not significantly correlated [13].

Different from the previously mentioned studies, we assessed treatment satisfaction using a single-item global measure instead of a comprehensive, multidimensional instrument. The underlying assumption of patient-rated global assessments is that the patients will weigh all factors related to their disease and provide a response that reflects their perspective of the construct being measured. Capturing an overall judgment based on a single question is beneficial when the assessment is based on personal criteria that vary from patient to patient. This is the case with overall treatment satisfaction. In addition, a single-item global measure fulfills the need for patient-rated measures to be short, easy to complete, easy to interpret, and clinically meaningful (5).

A key strength of our study is the recruitment of a population-based cohort of young people with long-duration type 1 diabetes. This study focuses on young people at a challenging time in their lives. The participants came from all regions of Germany and were treated at hospitals and in doctor's offices with different structures and under different frameworks. This variation in the sample likely increases the validity of our data and conclusions. A limitation is that our study is not representative of all children, adolescents and young adults with type 1 diabetes in Germany. People with good HbA1c values and high treatment satisfaction are probably overrepresented. The study may therefore underestimate the risk factors for impaired treatment satisfaction. Furthermore, the observational nature of our data limits the interpretation of causality. However, our study may be useful for hypothesis generation and can serve as a starting point for planning intervention studies.

## Conclusions

In conclusion, our results indicate a close relationship between treatment satisfaction and HbA1c values. There is also evidence that the needs of girls and women should receive greater attention. Further in-depth studies are desirable to gain a better understanding of interrelationships and to further adapt diabetes treatment to patient needs.

## Supplementary Information

**Additional file 1**: **Table S1**. Relative risks for impaired treatment satisfaction in 2018-2019 associated with sex, age group, and HbA1c level compared with the reference group. **Table S2**. Relative risks for impaired treatment satisfaction in 2018-2019 associated with sex, age group, HbA1c level, and treatment satisfaction in 2015-2016 compared with the reference group. **Figure S1**. Sankey diagram of intraindividual changes in treatment satisfaction over a 3-year period among girls (N=337). **Figure S2**. Sankey diagram of intraindividual changes in treatment satisfaction over a 3-year period among boys (N=238). **Figure S3**. Sankey diagram of intraindividual changes in treatment satisfaction over a 3-year period among 14- to 17-year-olds (N=151). **Figure S4**. Sankey diagram of intraindividual changes in treatment satisfaction over a 3-year period among 18- to 21-year-olds (N=221). **Figure S5**. Sankey diagram of intraindividual changes in treatment satisfaction over a 3-year period among 22- to 25-year-olds (N=121). **Figure S6**. Sankey diagram of intraindividual changes in treatment satisfaction over a 3-year period among 26- to 30-year-olds (N=82).

## Data Availability

The datasets used during the current study are available from the corresponding author on reasonable request.

## Abbreviations

HbA1c: Hemoglobin A1c
PRO: Patient-reported outcome
SD: Standard deviation
RR: Relative risk
95% CI: 95% Confidence interval
