# Supplementary material for: Increased risk of impaired treatment satisfaction among girls/women and young people with suboptimal HbA1c: Results of a nationwide type 1 diabetes study
Source: Diabetol Metab Syndr. 2021 May 19;13:55. doi: 10.1186/s13098-021-00673-0 (PMC8135928; doi:10.1186/s13098-021-00673-0)
Supplement: Supplementary file 1 — Additional file 1: Table S1. Relative risks for impaired treatment satisfaction in 2018-2019 associated with sex, age group, and HbA1c level compared with the reference group. Table S2. Relative risks for impaired treatment satisfaction in 2018-2019 associated with sex, age group, HbA1c level, and treatment satisfaction in 2015-2016 compared with the reference group. Figure S1. Sankey diagram of intraindividual changes in treatment satisfaction over a 3-year period among girls (N=337). Figure S2. Sankey diagram of intraindividual changes in treatment satisfaction over a 3-year period among boys (N=238). Figure S3. Sankey diagram of intraindividual changes in treatment satisfaction over a 3-year period among 14- to 17-year-olds (N=151). Figure S4. Sankey diagram of intraindividual changes in treatment satisfaction over a 3-year period among 18- to 21-year-olds (N=221). Figure S5. Sankey diagram of intraindividual changes in treatment satisfaction over a 3-year period among 22- to 25-year-olds (N=121). Figure S6. Sankey diagram of intraindividual changes in treatment satisfaction over a 3-year period among 26- to 30-year-olds (N=82). [file 13098_2021_673_MOESM1_ESM.docx]

**Supplemental Material**

**Increased risk of impaired treatment satisfaction among girls/women and young people with suboptimal HbA1c: Results of a nationwide type 1 diabetes study**

**Table S1** Relative risks for impaired treatment satisfaction in 2018-2019 associated with sex, age group, and HbA1c level compared with the reference group

| Variable |  | RR (95% CI) * | p value ^†^ |
| --- | --- | --- | --- |
| Sex | Girls/women vs boys/men | 1.64 (1.10 - 2.44) | 0.016 |
| Age group | 18 - 21 years vs 14 - 17 years | 0.75 (0.47 - 1.19) | 0.223 |
|  | 22 - 25 years vs 14 - 17 years | 0.90 (0.54 - 1.51) | 0.693 |
|  | 26 - 30 years vs 14 - 17 years | 1.58 ( 0.99 - 2.53) | 0.056 |
| HbA1c level | Unknown vs <7.5 % | 2.39 (0.97 - 5.89) | 0.058 |
|  | ≥7.5 % vs <7.5 % | 2.43 (1.63 - 3.63) | <0.001 |

* RR: Relative risk with 95% confidence interval from the log-binomial model

^†^ p value of the likelihood ratio test

**Table S2** Relative risks for impaired treatment satisfaction in 2018-2019 associated with sex, age group, HbA1c level, and treatment satisfaction in 2015-2016 compared with the reference group

| Variable |  | RR (95% CI) * | p value ^†^ |
| --- | --- | --- | --- |
| Sex | Girls/women vs boys/men | 1.40 (0.94 - 2.09) | 0.098 |
| Age group | 18 - 21 years vs 14 - 17 years | 0.78 (0.50 - 1.21) | 0.262 |
|  | 22 - 25 years vs 14 - 17 years | 0.86 (0.53 - 1.39) | 0.544 |
|  | 26 - 30 years vs 14 - 17 years | 1.22 (0.81 - 1.85) | 0.343 |
| HbA1c level | Unknown vs <7.5 % | 2.09 (0.92 - 4.72) | 0.078 |
|  | ≥7.5 % vs <7.5 % | 2.07 (1.40 - 3.06) | <0.001 |
| Treatment satisfaction in 2015-2016 | Impaired vs high | 2.77 (1.96 - 3.92) | <0.001 |

* RR: Relative risk with 95% confidence interval from the log-binomial model

^†^ p value of the likelihood ratio test


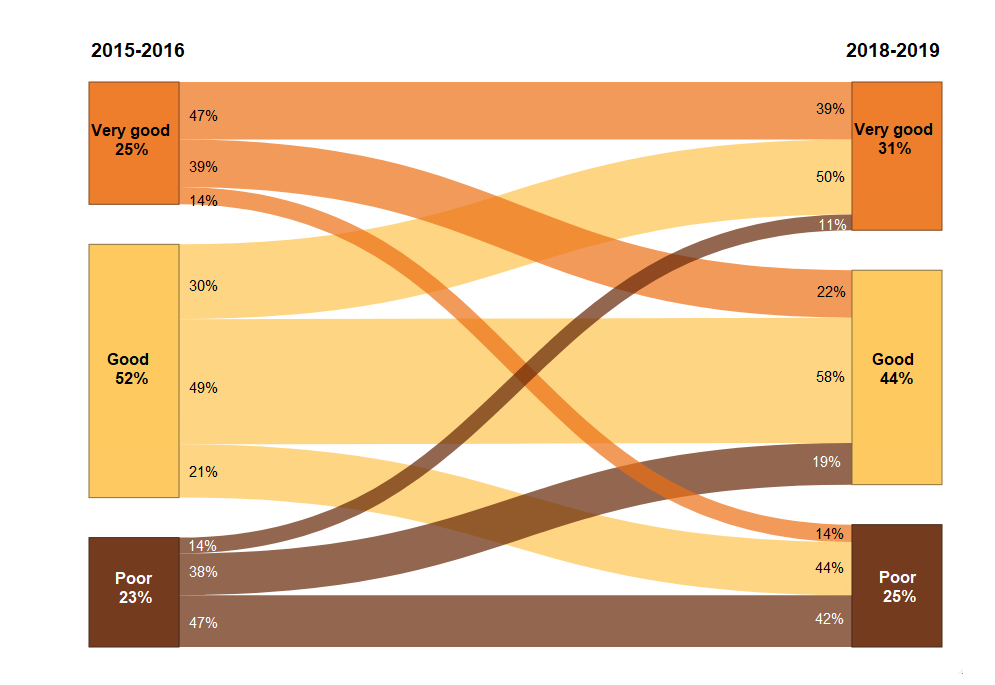
Figure S1: Sankey diagram of intraindividual changes in treatment satisfaction over a 3-year period among girls (N=337)


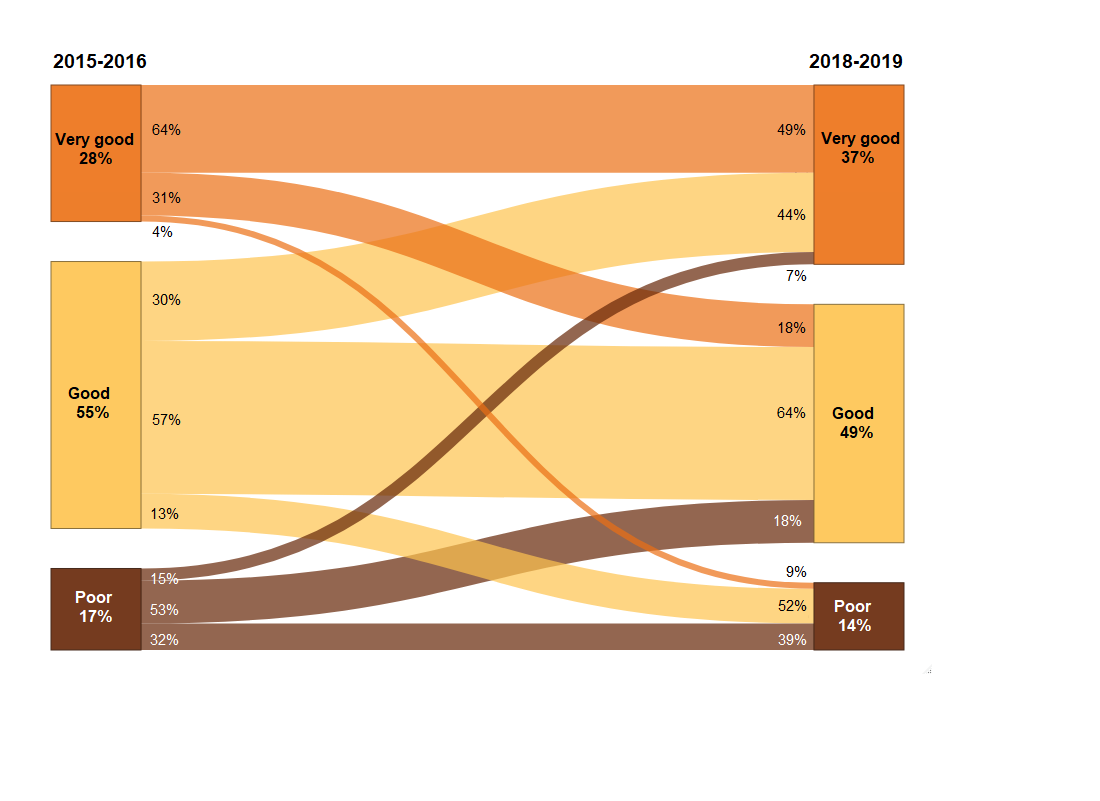


Figure S2: Sankey diagram of intraindividual changes in treatment satisfaction over a 3-year period among boys (N=238)


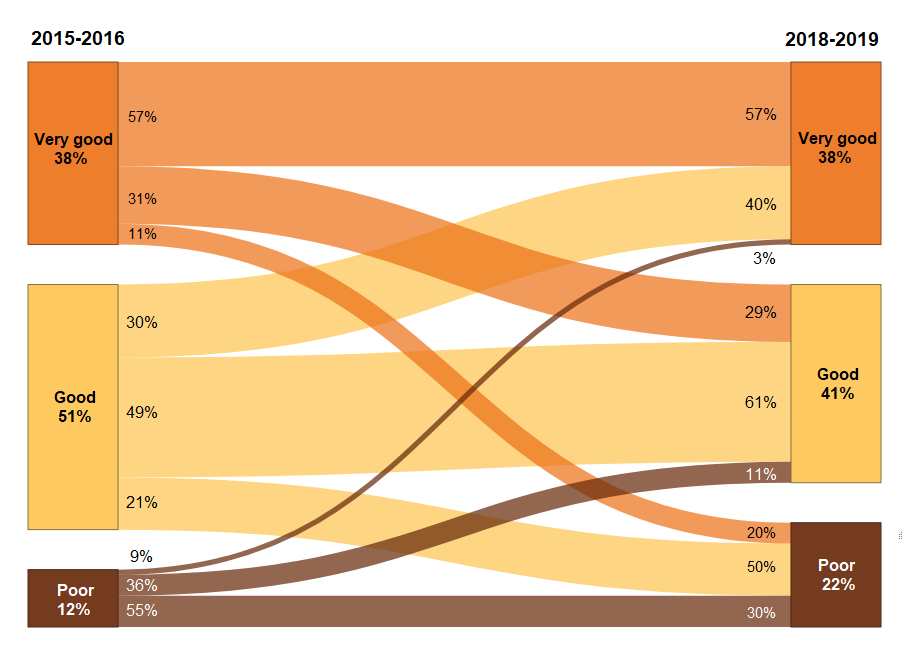


Figure S3: Sankey diagram of intraindividual changes in treatment satisfaction over a 3-year period among 14- to 17-year-olds (N=151)


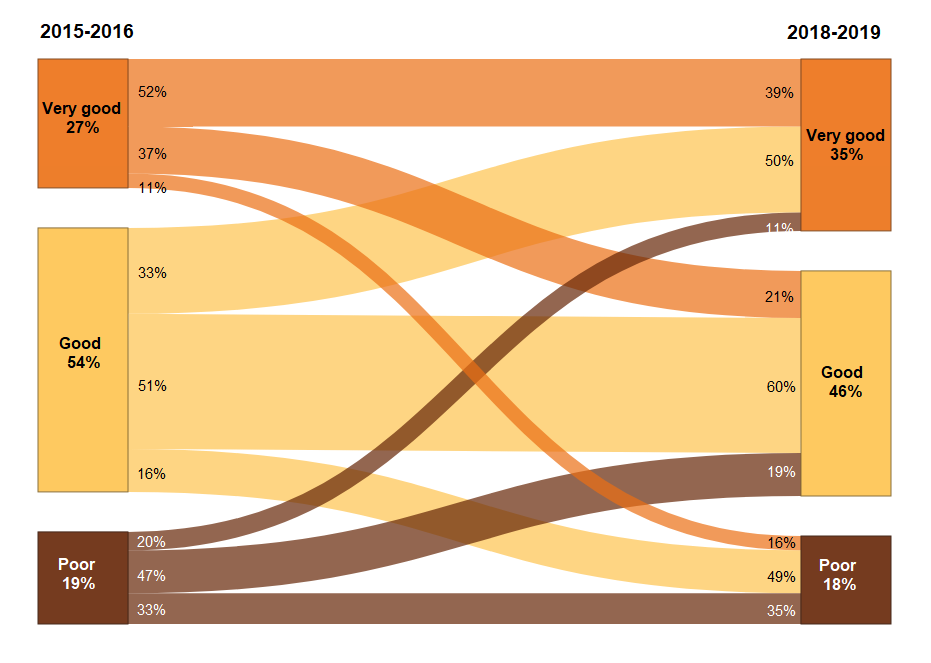


Figure S4: Sankey diagram of intraindividual changes in treatment satisfaction over a 3-year period among 18- to 21-year-olds (N=221)


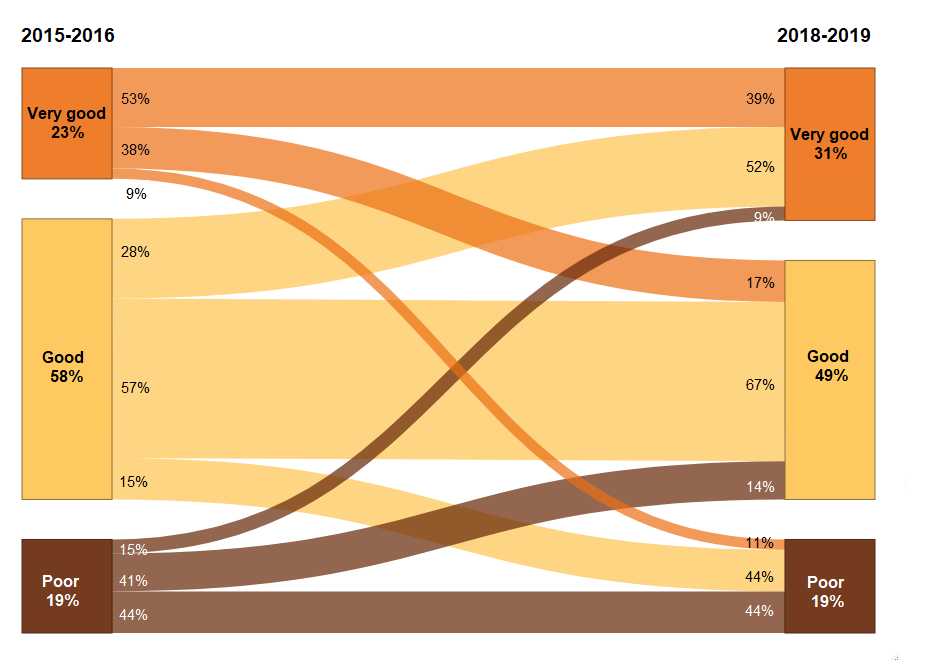


Figure S5: Sankey diagram of intraindividual changes in treatment satisfaction over a 3-year period among 22- to 25-year-olds (N=121)


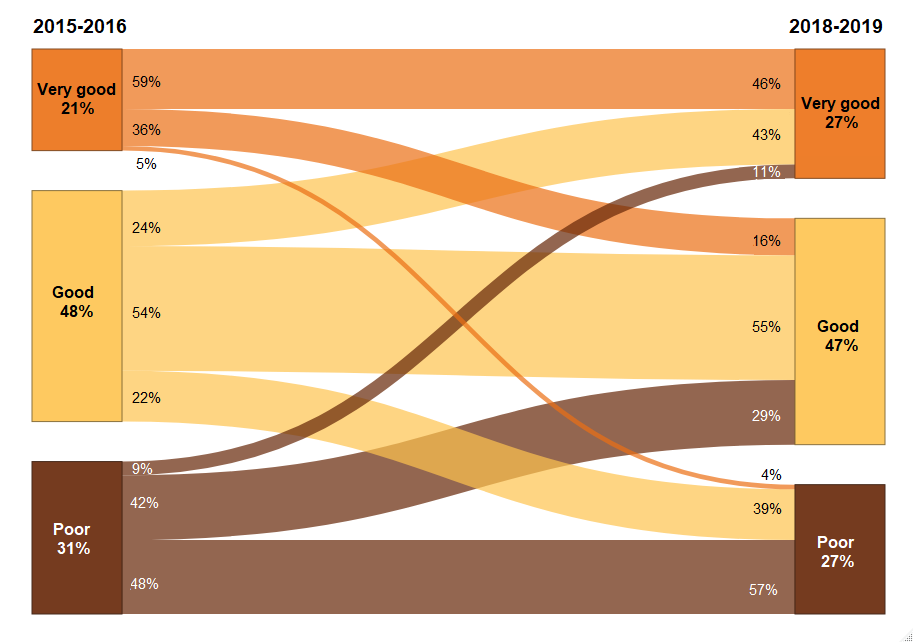


Figure S6: Sankey diagram of intraindividual changes in treatment satisfaction over a 3-year period among 26- to 30-year-olds (N=82)
